# Global Health Partnerships and the Brocher Declaration: Principles for Ethical Short-Term Engagements in Global Health

**DOI:** 10.5334/aogh.3577

**Published:** 2022-05-17

**Authors:** Shailendra Prasad, Myron Aldrink, Bruce Compton, Judy Lasker, Peter Donkor, David Weakliam, Virginia Rowthorn, Efua Mantey, Keith Martin, Francis Omaswa, Habib Benzian, Erwin Clagua-Guerra, Emilly Maractho, Kwame Agyire-Tettey, Nigel Crisp, Ramaswami Balasubramaniam

**Affiliations:** 1 University of Minnesota, US; 2 Medical and Surgical Skills Institute, GH; 3 Catholic Health Association, US; 4 Lehigh University, US; 5 Kwame Nkrumah University of Science and Technology, GH; 6 Health Services Executive, IE; 7 University of Maryland, US; 8 University of Ghana, GH; 9 Consortium of Universities of Global Health, US; 10 African Center for Global Health and Social Transformation, UG; 11 College of Dentistry, New York University, US; 12 Universidad de San Carlos de Guatemala, GT; 13 Ugandan Christian University, UG; 14 All Party Parliamentary Group on Global Health, UK; 15 Grassroots Research and Advocacy Moverment, IN

## Abstract

Short- term experiences in global health (STEGH), also known as short-term medical missions continue to be a popular mode of engagement in global health activities for students, healthcare providers, and religious groups, driven primarily by organizations from high-income countries. While STEGH have the potential to be beneficial, a large proportion of these do not sustainably benefit the communities they intend to serve, may undermine local health systems, operate without appropriate licenses, go beyond their intended purposes, and may cause harm to patients. With heightened calls to “decolonize” global health, and to achieve ethical, sustainable, and practical engagements, there is a need to establish strong guiding principles for global health engagements. The Advocacy for Global Health Partnerships (AGHP), a multi-sectoral coalition, was established to reflect on and address the concerns relating to STEGH. Towards this end, AGHP created the *Brocher Declaration* to lay out six main principles that should guide ethical and appropriate STEGH practices. A variety of organizations have accepted the Declaration and are using it to provide guidance for effective implementation of appropriate global health efforts. The Declaration joins broader efforts to promote equity in global health and a critical reevaluation of volunteer-centric, charity-based missions. The current state of the world’s health demands a new model of collaboration – one that sparks deep discussions of shared innovation and builds ethical partnerships to address pressing issues in global health.

## Introduction

In recent years, educational institutions, healthcare providers, non-government organizations (NGOs), and religious groups have increased the scope and practice of healthcare-related activities for short periods of time abroad [1]. These activities, referred to in this paper as short term experiences in global health (STEGH) but sometimes called short-term medical missions, are popular amongst a wide range of social groups including; students (high school, pre-healthcare, various health profession students, post-graduate trainees, etc.), religious groups, healthcare professionals, corporate leaders, and others. While organizational and individual motives for such engagements vary [2,3], the more pertinent discussion should be around the actual conduct and impact of such programs. To facilitate critical reflection on the conduct and impact of STEGH, there is a need for clearly stated principles to frame the ethical issues in this field and buttress regulatory frameworks for involved countries and organizations. We describe the development, dissemination, and implementation of the *Brocher Declaration*, a set of guiding principles for organizations to improve their global health practices to reduce harm, protect patients, increase benefits for host communities, reduce inequities and address power imbalances implicit in current STEGH [4,5].

To date, there are no universal, authoritative, or widely-accepted best practice guidelines for STEGH [6]. Although many stakeholders have created guidelines, they are limited by lack of regulatory structures or enforcement, either in host or sending countries, leading to the current situation in which STEGH can operate under no standards or accountability [7]. Unfortunately, in too many cases, this has been to the detriment of the communities in which these STEGH operate [1]. Despite potential and acknowledged benefits [8], many host communities are frustrated or dismayed with the negative effects that STEGH may have on local healthcare systems [4,9]. There are also deep concerns regarding the competency and authority of volunteers to perform needed tasks [7,8]. The arrival of international participants masquerading as “experts” may undermine local staff, even if the “experts” are high school students inappropriately garbed in medical scrubs [5]. Host communities have become more vocal in sharing their experiences regarding STEGH participants who are not professionally qualified, locally licensed, culturally sensitive, or respectful of local expertise [5,10,11]. In addition to these concerns, a fundamental question related to the cost-effectiveness and cost-benefit of STEGH remains to be addressed – could the significant investment individuals make to participate in these programs, estimated to be about US $ 3.7 billion a year from the US alone [12], be better utilized to strengthen local healthcare systems, expand training of health care professionals, build public health structures and accessible, appropriate and sustainable universal healthcare? Although “helping” host communities is cited as the overwhelming goal behind STEGH, the focus on the volunteer experience and limited attention to long-term and sustainable improvement of health care systems suggests that the benefits favor the visiting volunteers over host communities, perpetuating exploitative colonial patterns [4,5].

Calls to develop principles for STEGH programs are part of a bigger conversation around the need to decolonize global health practice, research, and education [13,14]. STEGH have been called out as a symptom or reflection of a colonizing mindset by exploiting power differentials to create volunteer opportunities for high income country individuals with limited regard for the needs of communities that host STEGH [15,16]. An essential element in this is ‘decolonizing the mind’ where creating a new antiracist mindset is a prerequisite to doing the structural work – thereby dismantling the structural drivers of discrimination [14,17]. As such, there have also been calls for “closing the door to parachutes and parasites” to ensure that such that the work in global health is not extractive but rather mutually beneficial and grounded in ethical principles [18].

To operate effectively and ethically, it is crucial for visitors from high-income countries to question the assumptions that underpin STEGH and actively shift the paradigm from “helping” and “giving/giving back” to “learning” and “sharing” [5,19]. Additionally, STEGH must incorporate the most effective and equitable use of often limited resources while supplementing or building the host community’s health workforce so that community needs can be addressed more sustainably.

## Principle Development Process

Advocacy for Global Health Partnerships (AGHP) has its origins in a side event at the Consortium of Universities for Global Health’s (CUGH) 2017 annual conference [20]. AGHP includes representatives from health professions and many other sectors from around the world involved in STEGH—faith-based, NGOs, corporate, and academic organizations in the Global North, and in host communities – all with relevant experience and a passion for improving STEGH. It was the first opportunity to find common ground across what are usually siloed activities, and to commit to continuing the conversation and carrying out research, education, and advocacy to advance shared goals.

An initial research effort involved a review of existing guidelines to identify common themes. Several meetings with various groups involved in STEGH followed and helped articulate the challenges related to STEGH. Emerging consensus indicated that the lack of regulatory mechanisms and the absence of strong voices from host countries were major bottlenecks to developing and implementing STEGH guidelines, reducing them to academic exercises with limited impact [6]. AGHP pursued further research, contributing to an analysis of the legal implications of common STEGH activities [21], and launched studies in three host countries -Ghana, Uganda and Guatemala – to gain a clearer perspective on host communities’ views and regulatory frameworks.

AGHP was awarded a grant from the Brocher Foundation in Geneva, Switzerland to hold a meeting of global health leaders, originally planned for May 2020, to address the goals identified above, to issue a unifying document with the key principles to guide short-term engagements, and to discuss strategies for their implementation. Following the COVID-19 related cancellation of that meeting, a series of consultations with the expected attendees and global health leaders representing thirty-six agencies, led to the creation of the “Brocher Declaration” [22].

## Principles

The Brocher Declaration is based on six foundational principles (see ***Table 1***) to guide STEGH towards more appropriate, equitable, sustainable, and ethical practices:

**Table 1:** Six Principles of the Brocher Declaration.


*1)* Mutual partnership with bidirectional input and learning *–emphasize mutual partnership and bidirectionality–both parties have input and learn from one another*. *–recognize expertise and experience of host country health professionals*. *–establish equality, trust and partnership as the foundations of all activities*

*2)* Empowered host country and community define needs and activities *–create programs based on the host country and community’s priorities* *–define activities such that external actors do not divert funds and efforts from real needs of the community* *–align with national planning frameworks and WHO/SDG priorities*

*3)* Sustainable programs and capacity building *–commit to long-term healthcare development and sustainability* *–aim to strengthen health systems rather than providing unsustainable alternatives* *–emphasize and utilize existing health systems*

*4)* Compliance with applicable laws, ethical standards, and code of conduct *–comply with existing legal and regulatory frameworks in the host and originating countries and with local regulations for professional practice and drug distribution* *–consider ethical principles including social justice, social contract, and utilitarian principles* *–abide by common quality principles*

*5)* Humility, cultural sensitivity, and respect for all involved *–respect the culture, history, strengths, expertise, and knowledge of host communities* *–recognize the limitations of visitors’ cursory understanding as non-members of the community and that they are subject to the constraints and biases of their own cultural backgrounds* *–transform the current narrative of privileged volunteers gaining social capital with lower regard for the perspectives of the host communities to one of solidarity and respect*

*6)* Accountability for actions *–evaluate programs appropriately so that negative outcomes and unintended consequences are reduced* *–place special emphasis on the concerns of environmental impact due to the travel and activities involved*. *–ensure accountability to local authorities*


### 1) Mutual partnership with bidirectional input and learning

*Health care varies greatly in terms of diseases, cultural and social determinants of health, languages spoken, clinical protocols, as well as political and economic conditions. This often leads to misalignment of short-term global health activities with the host country workforce and health priorities. Global health engagement should emphasize mutual partnership and bidirectionality, recognizing the expertise and experience of host country health professionals*.

STEGHs are often inappropriate or ineffective due to lack of consideration of the host community’s needs and capabilities [23]. Equality, trust and partnership should be the foundations for all global health engagements [15]. There should be a concerted effort to establish structures and practices that allow for communication with and involvement of host communities to identify needs, concerns, expertise, and experience. STEGH activities should strengthen bidirectional partnerships in which both parties have input and learn from one another [24].

### 2) Empowered host country and community define needs and activities

*When short-term global health engagements are based on perceived needs or available skills, they can undermine the local voice while diverting much needed funds and efforts away from real needs, along with placing added burden of accommodation and safety on host communities. This can be exacerbated by power differentials between people in high- and low-income countries. The host country should drive the agenda for healthcare work. This begins with empowered host communities who understand specific needs for health care and indicate the activities that would lead to sustained health improvement. Special emphasis should be placed on the social determinants of health and the relevant Sustainable Development Goals (SDG)*.

Often, STEGHs establish goals for their activities without consultation or consent of host community members [4,25]. Defining activities based on externally perceived needs or available skills diverts much needed funds and efforts away from communities’ real needs. The host country should drive the agenda for healthcare work, with special emphasis on relevant, long-term, system-level health improvements, training, and improving the social determinants of health in line with national planning frameworks and their respective objectives.

### 3) Sustainable programs and capacity building

*Global health programs should aim at capacity building within local communities such that important health needs are met and strengthened. This is possible when programs have sufficient input from the local communities and are committed to long-term healthcare development and sustainability. The overarching goal should be one of strengthening health systems rather than providing unsustainable alternatives.*

STEGH, particularly ones that are driven by sending country priorities and perspectives, can be disruptive and be a drain on resources in host countries [11,13]. Moreover, if these are not done under a framework of local capacity building and are not sustainable, they may create a dependency structure and provide no long term benefit [26,27]. Capacity building in this context means understanding the local community’s workforce needs and devoting specific resources to achieve these. STEGH need to emphasize and utilize existing health systems rather than providing unsustainable alternatives, thus optimizing both the host community’s/institution’s and the participants’ resources.

### 4) Compliance with applicable laws, ethical standards, and code of conduct

*Quite often, short-term engagements do not consider the existing legal framework in the host country. Clinical care has been framed within the context of the classic bioethical principles of autonomy, justice, beneficence, and non-maleficence. Engaging in global health activities requires entities to consider other ethical principles including social justice, social contract, and utilitarian principles. Short-term global health partnerships must establish and abide by common quality principles and legal requirements*.

A major concern with STEGH programs is ignorance or subversion of relevant laws that do exist, for instance medical licensure regulations in host countries [17]. STEGH must comply with existing legal and regulatory frameworks in the host and originating countries and with local regulations for professional practice and drug distribution, among others. Besides classic bioethical principles of autonomy, justice, beneficence, and non-maleficence, STEGH need to ensure that they operate on sound public health and global health ethics [28].

### 5) Humility, cultural sensitivity, and respect for all involved

*International health volunteers and the organizations that coordinate their work often have motivations other than contributing to the health of people in host communities. These experiences can be seen as privileged volunteers gaining social capital at the expense of disadvantaged host communities. To alleviate this dynamic, those participating in short-term engagements must respect the culture, history, strengths, and limitations of the communities they are visiting, while simultaneously recognizing the limitations of their cursory understanding as non-members of the community*.

STEGH may at times be driven by motivations other than contributing to the health of people in host communities [2]. Unfortunately these motivations are often not examined and, in fact, actively buried under the predominant charity narrative that bolsters STEGH. The current narrative, and frequent focus of criticism, is that of privileged volunteers gaining social capital with lower regard for the perspectives of the host communities [9]. To change this narrative, those participating in STEGH acknowledge their own limitations, and approach the experience with a desire to learn from and work together with host country stakeholders. They should also recognize that their values and behaviors are subject to the constraints and biases of their own cultural backgrounds.

### 6) Accountability for actions

*The overall emphasis of global health engagements should be on long-term health improvement of host communities. Global health engagements should be evaluated appropriately so that outcomes, unintended consequences, and spillover effects are reduced. If these standards are not upheld by short-term global health engagements, or if they cause negative impacts, they should be altered or ended. There should be special emphasis placed on the concerns of environmental impact due to the travel and activities involved.*

Currently, no system exists to ensure that STEGH participants are performing their activities ethically and effectively [5,7]. This creates a space for volunteers to perform tasks they are unqualified to do or would not be able to perform in their home country. Host communities have the right to safety and efficacy as they open their facilities to volunteers [11]. STEGH should be evaluated appropriately to minimize adverse outcomes, learn from good experiences, monitor for unintended consequences, and negative spillover effects. Further, the reach of the laws of sending countries generally does not extend into host countries where violations take place, and local authorities often do not have the resources to pursue malpractice. As such, a multi-pronged approach, from both sending and host countries is needed to ensure accountability. Of note, with major concerns of the impact of travel across long distances and its contribution to climate change, STEGH should consider alternative models on engagements including connecting with partners virtually.

## Dissemination and Implementation

Since its release in 2020, AGHP has been actively disseminating the Brocher Declaration through a series of online primer sessions engaging various groups. The Brocher Declaration has been featured in various webinars and conferences [29,30,31,32,33]. It has also been endorsed by over 50 organizations from around the world and from multiple sectors [34]. It has been cited by several organizations as a valuable tool in reevaluating their practices, which was one of the key goals of establishing such guidance [5,35,36,37,38,39]. The declaration has been cited as a guide to educational and planning efforts in global health activities [37].

AGHP has been conducting quarterly networking calls with interested organizations and has also prompted primary research on the effect of STEGHs in individual countries. Future work in improving global health partnership would include regulatory and enforcement practices in countries around the world. AGHP will use the Brocher Declaration as a guiding principle to inform such activities.

## Conclusion

Global Health activities should aim to reduce disparities in health and improve wellbeing around the world [40]. While STEGH are popular in high-income countries, they are often wasteful and sometimes cause more harm than good to the host communities involved. Further, the current orientation of STEGH to “help” instead of learn perpetuates a misguided and colonial power dynamic that closes participants’ eyes to host country ways of seeing and doing that could benefit high income countries.

The COVID19 pandemic has brought STEGH to a halt, providing an opportunity to review the frameworks of global health engagements. We propose the Brocher Declaration as a starting point for stakeholders to focus their work on addressing power imbalances, risk of potential harm, and unethical practice that may be involved in their work. In the absence of a single regulatory process for STEGH, it is the responsibility of the participants and sending organizations to uphold these values, ethical principles, and applicable laws. We also hope it will be a springboard for reevaluations of volunteer-centric, charity-based missions when the current state of the world’s health demands a new model of collaboration – one that seeks the removal of structural, political, and economic barriers to health equity and fosters a shared responsibility for pressing issues in global health.
